# A novel microbial technique for producing high‐quality sophorolipids from horse oil suitable for cosmetic applications

**DOI:** 10.1111/1751-7915.13297

**Published:** 2018-07-18

**Authors:** Yoojae Maeng, Kyoung Tae Kim, Xuan Zhou, Litai Jin, Ki Soo Kim, Young Heui Kim, Suyeon Lee, Ji Ho Park, Xiuyu Chen, Mingxia Kong, Lu Cai, Xiaokun Li

**Affiliations:** ^1^ School of Pharmaceutical Sciences Wenzhou Medical University Wenzhou 325035 China; ^2^ Collaborative Innovation Center of Biomedicine Wenzhou Medical University‐Wenzhou University Wenzhou 325035 China; ^3^ Ningbo First Hospital Ningbo 315000 China; ^4^ BiolandBiotec. Co., Ltd. Zhangjiang Modern Medical Device Park Pudong, Shanghai 201203 China; ^5^ SK Bioland 59, Songjeongni 2‐gil, Byeongchen, Dongnam, Cheonan Chungnam 31257 Korea; ^6^ SK Bioland 162, Gwahaksaneop 3‐ro, Ochang Cheongwon, Cheongju, Chungbuk 28125 Korea; ^7^ Departments of Pediatrics, Radiation Oncology, Pharmacology and Toxicology Pediatric Research Institute University of Louisville Louisville KY 40202 USA

## Abstract

Horse oil contains linoleic, palmitoleic and unsaturated fatty acids that are similar to those in human skin, and may therefore be an ideal substance from which to isolate biosurfactants for cosmetic products to improve human skin quality. Herein, an innovative approach was developed to synthesise sophorolipids from horse oil by hydrolysis, followed by fermentation using the yeast *Candida bombicola*. The yield of sophorolipids from direct fermentation of horse oil and hydrolysed horse oil was 40.6 ± 1.3 g l^−1^ and 58.4 ± 1.8 g l^−1^ respectively. To further increase the yield, 30–40 g l^−1^ glucose was added in a fed‐batch fermentation process to maintain the pH between 4.0 and 4.5, resulting in a conversion yield of 71.7 ± 0.8 g l^−1^. The purity and structure of the synthesised sophorolipids were analysed by ultra‐performance liquid chromatography‐mass spectrometry and nuclear magnetic resonance. An *in vitro* human dermal fibroblast model was used as a surrogate for human skin to measure elastase inhibition activity. Antiwrinkle properties of isolated sophorolipids were better than those of horse oil or hydrolysed horse oil in several *in vitro* assays. Furthermore, no cytotoxicity was observed at a concentration of 50 μg ml^−1^, and wound‐healing capacity was evident in a cell culture model. Additionally, the synthesised sophorolipids attenuated lipopolysaccharide‐induced expression of inflammatory cytokines in macrophages, and efficiently inhibited several strains of bacteria and yeast. In conclusion, fed‐batch fermentation of hydrolysed horse oil is a novel and efficient approach for producing high‐quality and high‐yield sophorolipids that exhibit great potential as cosmetic ingredients.

## Introduction

Effective and safe (less toxic) ingredients are required for cosmetic products. The safety of cosmetics was first mentioned in the 1930s (Carleton, 1933), the effects of cosmetics on the skin barrier were first reported in 1946 and much research has since been published in this field (Sadler and Marriott, 1946). The production of biosurfactants by microorganisms was first described more than 90 years ago (Brown, 1991). Due to the societal norms of modern life, the use of cosmetic products has increased dramatically; the market for personal care products is expected to increase between 3.5% and 4.5% over the next 5 years, reaching a total market value of US $500 billion by 2020 (Adams, 2016). Furthermore, the market for specific product ingredients was valued at US$ 7.46 billion in 2014, and will likely grow even faster, reaching US $11.76 billion by 2023 (Adams, 2016).

Surfactants are amphiphilic molecules with both hydrophobic and hydrophilic regions. For this reason, they are adsorbed by, and arranged at, gas–liquid, liquid–liquid and liquid–solid interfaces, where they alter the surface characteristics. The adsorption of surfactants is related to their structure; hence, they are used for emulsifying, solubilising, dispersing, condensing, cleaning, absorbing moisture, permeating, foaming and lubricating. Researchers have developed and characterised surfactants with different properties, from traditional synthetic surfactants, to more recent biosurfactants (Van Bogaert *et al*., 2007; Daverey and Pakshirajan, 2009; Olkowska *et al*., 2014; Deng *et al*., 2016).

Biosurfactants are surface‐active compounds from biological sources that are usually produced extracellularly or as part of the cell membrane by bacteria, yeast and fungi. Based on the molecular structure and the extent of hydrophilic or hydrophobic regions, biosurfactants are categorised into several glycolipid types including sophorolipids, rhamnolipids and cellular biolipids, of which sophorolipids are the most abundantly produced (Baccile *et al*., 2016; Radzuan *et al*., 2017). Sophorolipids are composed of disaccharide sophorose fats. Glucose as a main carbon source can provide the sophorose group, and oils and fatty acids as secondary carbon sources provide the lipid chains. Recently, an improved method was reported for the production of acidic sophorolipids by methyl esterification of oils or fatty acids (Lourith and Kanlayavattanakul, 2009; Shin *et al*., 2010). Horse oil, a subcutaneous fat extracted from the herbivorous horse, is included on the Chinese cosmetics ingredient list. Linoleic acid, palmitoleic acid and unsaturated fatty acids are major components of horse oil, comprising 60% to 65% by weight, which is similar to human skin (Table S1), hence their suitability for use in cosmetic skin care products.

The present study aimed to improve the conversion yield of sophorolipids by hydrolysing horse oil and fermenting the products using the yeast *Candida bombicola* ATCC22214. Furthermore, the process was scaled up to demonstrate the ability to prepare large quantities for use in the cosmetics industry. In addition, antiwrinkle and anti‐inflammatory properties of the isolated sophorolipids were evaluated using several *in vitro* assays that are commonly employed for testing the feasibility of commercial production of ingredients for use in cosmetic products.

## Results and discussion

### Maximising the yield of sophorolipids by improving the extraction protocol, and the structure and compositions of synthesised sophorolipids

**Figure 1:**
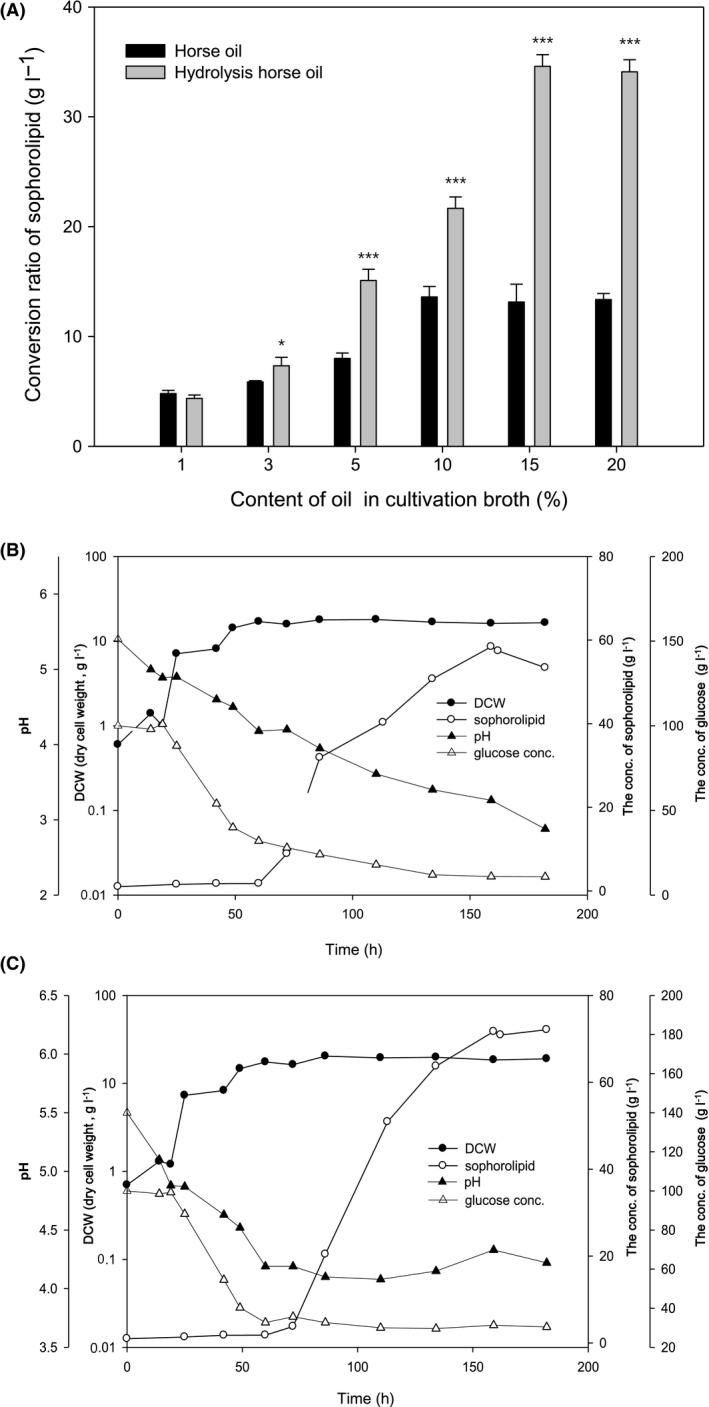
Yield of sophorolipids from horse oil and hydrolysed horse oil, and the effects of pH and glucose concentration on yeast growth and sophorolipid yield. To establish optimal conditions, horse oil was diluted with water to different concentrations in flask cultures and treated with 0.3% lipase at 35°C with stirring for 1 h, and sophorolipids were measured using the solvent extraction method (A). Data are presented as the mean ± SD from three separate experiments respectively. *p <* *0.05 or ***p <* *0.001 vs. corresponding horse oil group. Dynamic profiles of yeast growth status, evaluated by dry cell weight (DCW), sophorolipid concentrations, pH values and glucose concentrations in the 5 L jar fermenter were measured at different time points during routine fermentation (B) and fed‐batch fermentation (C) in which pH was maintained above 4 by frequent addition of glucose after 50 h of fermentation. Data are presented as the mean ± SD from three separate experiments respectively. *p <* *0.05 vs. controls (time = 0); **p <* *0.01 vs. controls (time = 0); ***p <* *0.001 vs. controls (time = 0).

To establish optimal experimental conditions, we diluted horse oil (purchased from BiolandBiotec. Co., Ltd., Haimen, Jiangsu, China) with water to different concentrations in culture flasks, added 0.3% lipase and incubated samples at 35°C with stirring for 1 h. Sophorolipids in flasks were measured using solvent extraction methods, and the sophorolipid yield was increased with increasing concentration of both substances (horse oil and lipase), reaching 13.6 ± 1.1 g l^−1^ when the concentration of horse oil was 10% or higher, and up to 34.6 ± 1.9 g l^−1^ with 15% or higher hydrolysed horse oil (Fig. 1A). These results suggest that the conversion yield of sophorolipids from hydrolysed horse oil was more than 2.5‐fold greater than that from untreated horse oil (Table 1).

**Table 1:** Conversion rate of sophorolipid from horse oil and hydrolysed horse oil at different conditions

Oil substance	Fermentation conditions	Conversion rate of sophorolipid (g l^−1^)
Horse oil	Flask culture, oil 10%	13.6 ± 1.1
Hydrolysed horse oil	Flask culture, oil 15%	34.6 ± 1.9
Horse oil	5 l fermenter batch culture, oil 10%	40.6 ± 1.3
Hydrolysed horse oil	5 l fermenter batch culture, oil 15%	58.4 ± 1.8
Hydrolysed horse oil	5 l fermenter fed‐batch fermentation, oil 15%	71.7 ± 0.8

*Candida bombicola* is capable of producing large quantities of biosurfactant sophorolipids (Roelants *et al*., 2013; Elshafie *et al*., 2015; Konishi *et al*., 2016; Samad *et al*., 2017). Therefore, to further optimise the procedure, hydrolysed horse oil was fermented with *C. bombicola* in a 5 l jar fermenter. Initially, there was no change in pH or glucose concentration during the fermentation period of 7 days. Horse oil or hydrolysed horse oil at 5% was added at 50, 100 and 150 h of fermentation, so that the final concentration in the fermenter was 10% for horse oil or 15% for hydrolysed horse oil. Figure 1B shows a representative fermentation prolife for hydrolysed oil, in which the yeast cell density, measured by dry cell weight (DCW), was sharply increased after 24 h of fermentation, and plateaued at a maximum level after 50 h. The conversion yield of sophorolipids from hydrolysed horse oil was significantly increased from 60 h onwards, and reached a peak of 58.4 ± 1.8 g l^−1^ after 160 h of fermentation (Fig. 1B, Table 1). Under similar conditions, we also observed an increase in the conversion yield of sophorolipids from horse oil with increasing fermentation time, reaching a peak yield of 40.6 ± 1.3 g l^−1^ (Table 1; fermentation profile not shown).

Figure 1B also reveals that, during fermentation, the glucose concentration began to quickly decrease after 24 h of fermentation, from the original 100 g l^−1^ to < 10 g l^−1^, and reached almost 0 g l^−1^ after 50 h (Fig. 1B). Additionally, the pH gradually decreased throughout, from pH 5.4 at the start of fermentation, to pH ~2.8 at the end (180 h; Fig. 1B). Sophorolipids are produced in large quantities during the latter phase of exponential growth, or during the stationary phase when carbon or nitrogen is in short supply (Ma *et al*., 2011). Thus, addition of other matrix types (hydrocarbons and oils) during these phases can increase production substantially. Sophorolipids are composed of disaccharide sophorose fats, and glucose as a main carbon source can provide the sophorose group, while oils and fatty acids as secondary carbon sources can provide the lipid chains. In addition, glucose is required for both the growth of *C. bombicola* and sophorolipid conversion. In fact, the existence of a stationary phase for cell growth between 24 and 50 h of fermentation also suggests that further glucose consumption may be required predominantly for sophorolipid conversion from 50 h onwards. Therefore, to improve conversion of sophorolipids in the 5 l fermenter, 5% hydrolysed horse oil was added at 50, 100 and 150 h, to achieve a final concentration of 15% as described above, but from 50 h the pH was adjusted to ~4.0–4.5, and glucose was added to maintain the concentration range between 30 and 40 g l^−1^ (Fig. 1C). These modifications (i.e., fed‐batch fermentation) resulted in a significant increase in the sophorolipid conversion yield to 71.7 ± 0.8 g l^−1^ after fermentation for 180 h (Fig. 1C), which is ~5.3‐ and 2.0‐fold higher than the yield achieved with horse oil and hydrolysed horse oil, respectively, without fed‐batch fermentation (Table 1).

Therefore, through the optimisation process, we conclude the following: (i) the optimal concentration of horse oil in the cultivation broth was 10%, but 15% hydrolysed horse oil was optimal for generating sophorolipids (Fig. 1A); (ii) the conversion rate of hydrolysed horse oil to sophorolipids was higher than that of untreated horse oil (in small‐scale experiments); (iii) the conversion rate of fed‐batch fermentation was higher than that of bath fermentation. These results represent an improvement over those of a previous study that optimised the production of C12–C14 sophorolipids from coconut oil using *C. bombicola* in a fed‐batch fermentation (Morya *et al*., 2013). The authors maintained a 10% glucose concentration and obtained a maximum sophorolipid yield of 54.0 g l^−1^ after 234 h of fermentation (Morya *et al*., 2013). By comparison, we achieved a maximum yield of 71.6 g l^−1^ after only 50 h of fermentation (Fig. 1C).

After fermentation, a mixture of lactonic and acidic sophorolipids was purified by solvent extraction followed by column chromatography. The structures of seven sophorolipids were defined by ultra‐performance liquid chromatography‐mass spectrometry (UPLC‐MS) analysis using the method outlined in Fig. S2A and B. The proportion of each type of sophorolipid and the molecular weight are listed in Table S2. Lactonic sophorolipids were more abundant, with diacetyl 18:1 accounting for 40.12% and diacetyl 18:0 for 22.78%, while the third most abundant was the acidic diacetyl C18:1 sophorolipid (17.69%).

**Figure 2:**
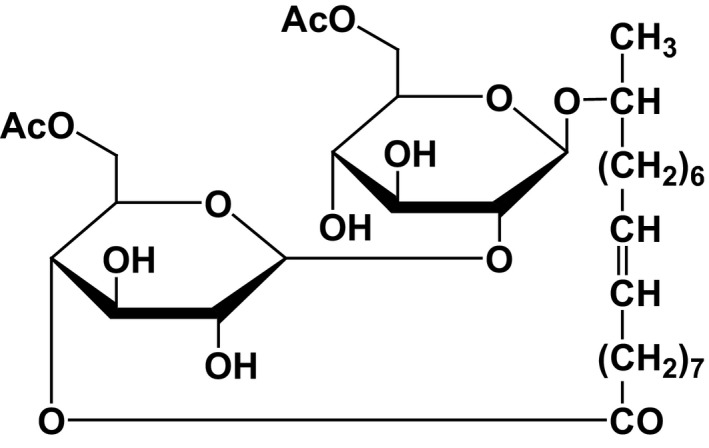
Structure of the isolated compound. Structural analysis of the most abundant sophorolipid (lactonic diacetyl C18:1) isolated from the mixture.

To confirm the structure of the most abundant species, the lactonic sophorolipid diacetyl 18:1 was isolated from the mixture, as shown in Fig. 2, and the isolated compound was subjected to nuclear magnetic resonance (NMR) spectroscopy using ^1^H‐NMR (MeOD, 600 MHz; Fig. S3A), ^13^C‐NMR (MeOD, 150 MHz; Fig. S3B), homonuclear correlation spectroscopy (COSY; Fig. S4), distortionless enhancement by polarisation transfer (DEPT; Fig. S5) and heteronuclear multiple bond correlation (HMBC; Fig. S6). We confirmed that the isolated compound was 9‐octadecenoic acid, with the structure 17‐[[6‐O‐acetyl‐2‐O‐(6‐O‐acetyl‐β‐D‐glucopyranosyl)‐β‐D‐glucopyranosyl]oxy]‐,intramol.1,4″‐ester,(9Z), consistent with previous reports (Hu and Ju, 2001; Morya *et al*., 2013) (Fig. S1).

### Biological evaluation of sophorolipids synthesised from horse oil

We investigated the biological activities of the synthesised sophorolipids using several *in vitro* assays to test their suitability for cosmetic purposes. The skin, a major organ that is capable of continual renewal, is responsible for various physiological functions such as protection against pathogens and UV radiation. Although visible, skin is similar to other nonvisible organs of the body, and is similarly subject to complex and cumulative aging processes throughout life. In general, aged skin exhibits an imbalance between adequate loss and replenishment of structural and functional components. Dermal aging may include dermal thinning due to decreases in the number of fibroblasts and collagen synthesis, increased UV‐induced collagen degradation, and loss of dermal elasticity due to dermal elastin network destruction caused by either reduced synthesis of elastin or increased degradation of elastic fibres, leading to the appearance of wrinkles (Langton *et al*., 2010; Samad *et al*., 2017; Weihermann *et al*., 2017).

#### Elastase inhibition and antiwrinkle activity

**Figure 3:**
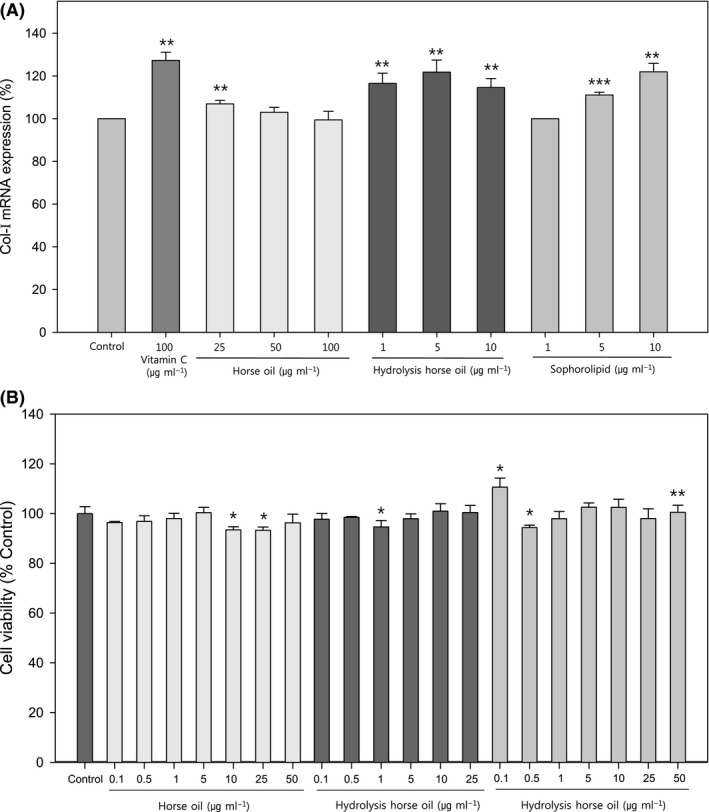
Effects of horse oil, hydrolysed horse oil and sophorolipids on collagen‐I mRNA expression and cell viability. Effects of different concentrations of horse oil, hydrolysed horse oil or sophorolipids on Col‐I mRNA expression (A) and cell viability (B) in human skin fibroblasts were determined along with the vitamin C‐treated group as a positive control (panel A). Data are presented as the mean ± SD from three separate experiments respectively. *P <* *0.05 vs. controls; **P <* *0.01 vs. controls; ***P <* *0.001 vs. controls.

The effects of sophorolipids on the capacity of human skin fibroblasts to express collagen I (Col‐I) mRNA were examined. Vitamin C (ascorbic acid) increases collagen type 1 mRNA expression in human fibroblasts to regulate skin elasticity (Kishimoto *et al*., 2013); hence, we used it as a positive control in the present study. Compared with controls, vitamin C at 100 μg ml^−1^ increased Col‐I mRNA expression by 127%, while horse oil did not have any stimulating effect on Col‐I mRNA expression, even at a concentration of 25–100 μg ml^−1^. However, both hydrolysed horse oil and sophorolipids stimulated Col‐I mRNA expression to a similar extent, and the effect was concentration‐dependent in the case of sophorolipids (Fig. 3A).

Since the level of elastin in the extracellular matrix (ECM) is determined by both its generation and degradation, the effect of sophorolipids on elastase activity was examined in cultured cells *in vitro* by measuring the elastase inhibition activity (IC_50_ value). Horse oil displayed no elastase inhibition activity, whereas hydrolysed horse oil exhibited potent elastase inhibition activity with an IC_50_ of 98.2 μg ml^−1^, and sophorolipids were even more potent with an IC_50_ of 38.5 μg ml^−1^ that is close to that of the positive control kaempferol (32.8 μg ml^−1^). Kaempferol is a polyphenol antioxidant and potent elastase inhibitor possessing free radical‐scavenging activity, and both actions are important for antiaging (Lee *et al*., 2010, 2015; Azmi *et al*., 2014); hence it was used as a positive control in the present study. To our knowledge, there are no previous reports providing *in vitro* assay evidence of the antiwrinkle effects of horse oil products.

#### Cytotoxicity and migration potential

To ensure the suitability of sophorolipids as cosmetic products, cytotoxicity must be ruled out. Therefore, we measured cell viability by thiazolyl blue tetrazolium bromide (MTT) assay and found no significant cytotoxicity of horse oil up to 50 μg ml^−1^, hydrolysed horse oil up to 25 μg ml^−1^ and sophorolipids up to 50 μg ml^−1^. Interestingly, sophorolipids at low levels (0.1 μg ml^−1^) had a stimulatory effect, but neither horse oil nor hydrolysed horse oil had such effects (Fig. 3B).

**Figure 4:**
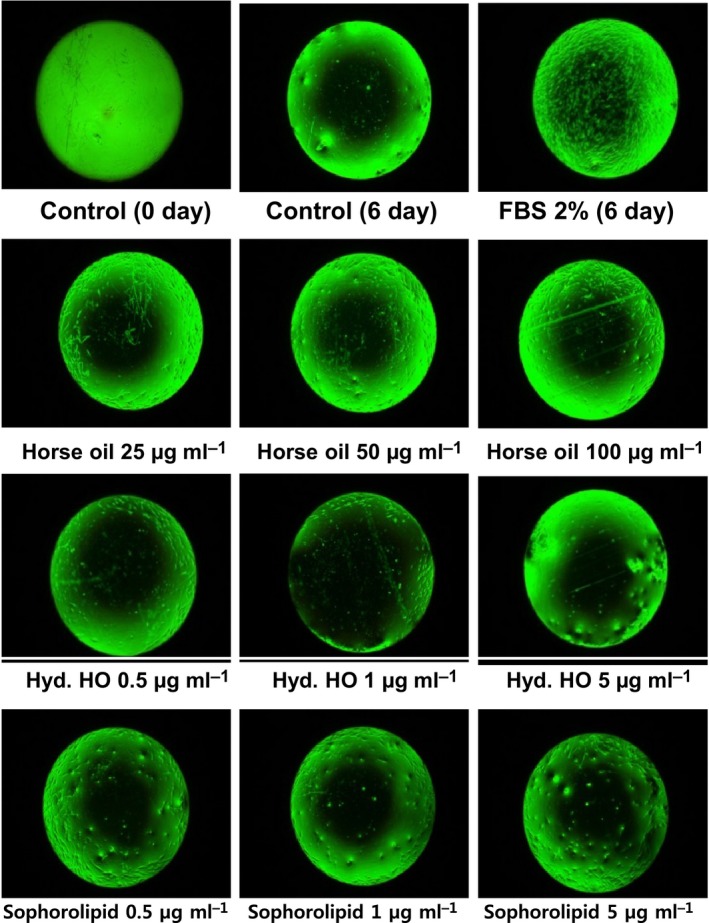
The effect of horse oil, hydrolysed horse oil or sophorolipid on wound‐healing potential measured using an *in vitro* cell culture model (cell migration assay). Effects of different concentrations of horse oil (25–100 μg ml^−1^), hydrolysed horse oil (0.5–5 μg ml^−1^) or sophorolipids (0.5–5 μg ml^−1^) on human skin fibroblast migration were examined using an Oris Cell migration assay kit. Fibroblasts were cultured at a density of 1 × 10^4^ cells/well in 96 well plates at 37°C for 24 h, the medium was substituted with serum‐free medium containing different concentrations of horse oil, hydrolysed horse oil (Hyd. HO) or sophorolipids, and culturing continued at 37°C for 6 days. After washing with 1× phosphate‐buffered saline (PBS), cells were observed by microscopy. Images are representatives of at least six samples of each group (duplicate samples in each experiment and three separate experiments respectively).

Epidermal cells form the outermost layer of skin, and they are constantly being shed and replaced by new cells generating beneath them. Fibroblasts are the most important cells for synthesising collagen and forming connective tissue; hence, they are crucial in wound healing and recovery. During primary intention wound healing, collagen synthesis and epidermal cell migration occur almost at the same time. To investigate the effects of sophorolipids on the wound‐healing potential, an *in vitro* cell culture wound‐healing model was established using an Oris Cell migration assay kit. Cultured cells were treated with sophorolipids and viewed under a microscope to examine the sizes of spaces filled and unfilled by cells (Fig. 4). The results revealed incomplete cell migration in groups treated with horse oil and hydrolysed horse oil, while a significant increase in human skin fibroblast migration was observed in the group treated with an appropriate concentration of sophorolipids.

#### Inhibitory effects on inflammatory cytokine expression and bacterial or fungal growth

**Figure 5:**
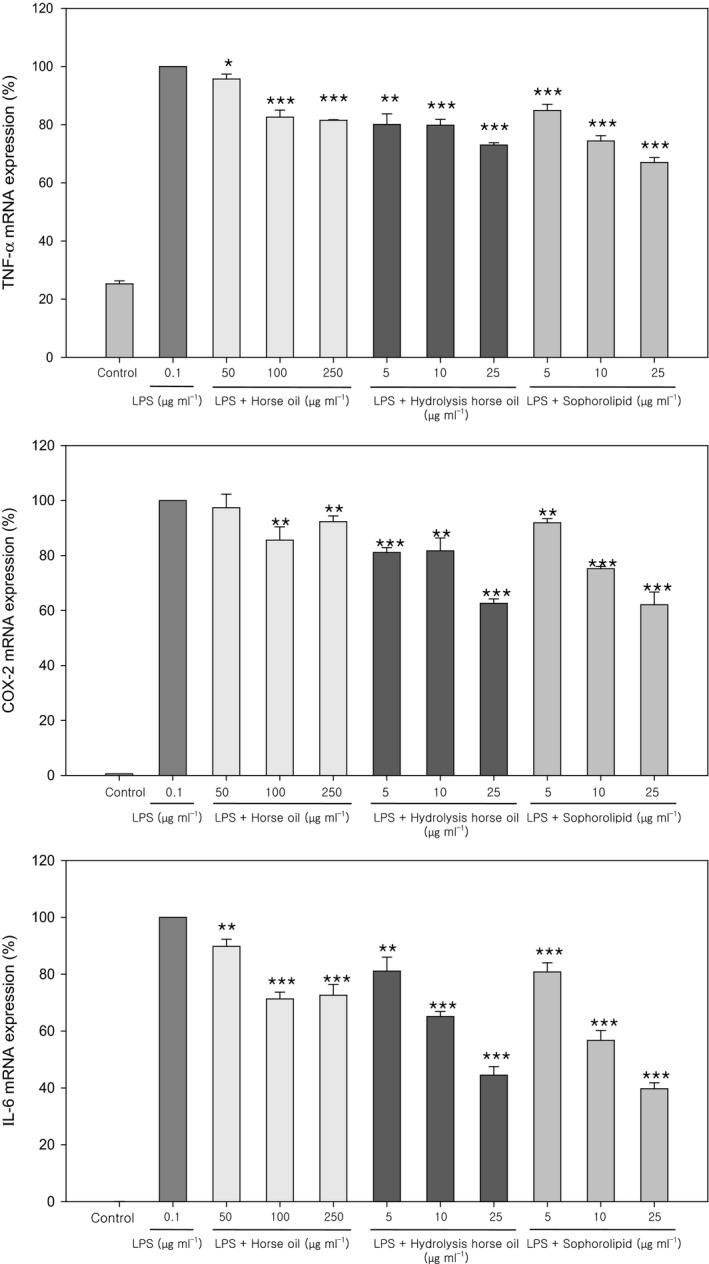
Anti‐inflammatory activity of horse oil, hydrolysed horse oil and sophorolipids measured as the decrease in lipopolysaccharide (LPS)‐induced miRNA expression of inflammatory cytokines. The effects of horse oil, hydrolysed horse oil or sophorolipids at the indicated concentrations on LPS‐induced mRNA expression of inflammatory cytokines (TNF‐α, COX‐2 and IL‐6) in mouse RAW 264.7 macrophages were examined by real‐time PCR. Data are presented as the mean ± SD from three separate experiments respectively. *P <* *0.05 vs. LPS (0.1 μg ml^−1^); **P <* *0.01 vs. LPS; ***P <* *0.001 vs. LPS.

Lipopolysaccharide (LPS) is an inflammatory substance that increases inflammatory mediators such as interleukins (ILs), tumour necrosis factor (TNF)‐α and nitric oxide synthase (iNOS). Therefore, we tested whether, under stimulation by LPS, sophorolipids could inhibit the transcription of inflammatory cytokines including TNF‐α, COX‐2 and IL‐6, using RT‐PCR (Fig. 5). LPS‐stimulated expression of TNF‐α, COX‐2 and IL‐6 mRNA in mouse macrophage RAW 264.7 cells was significantly inhibited by horse oil at concentrations of 100 μg ml^−1^ and higher. However, both hydrolysed horse oil and sophorolipids significantly inhibited the mRNA expression of TNF‐α, COX‐2 and IL‐6 induced by LPS within a concentration range of 5–25 μg ml^−1^, and in a clear concentration‐dependent manner in the case of sophorolipids (Fig. 5).

**Figure 6:**
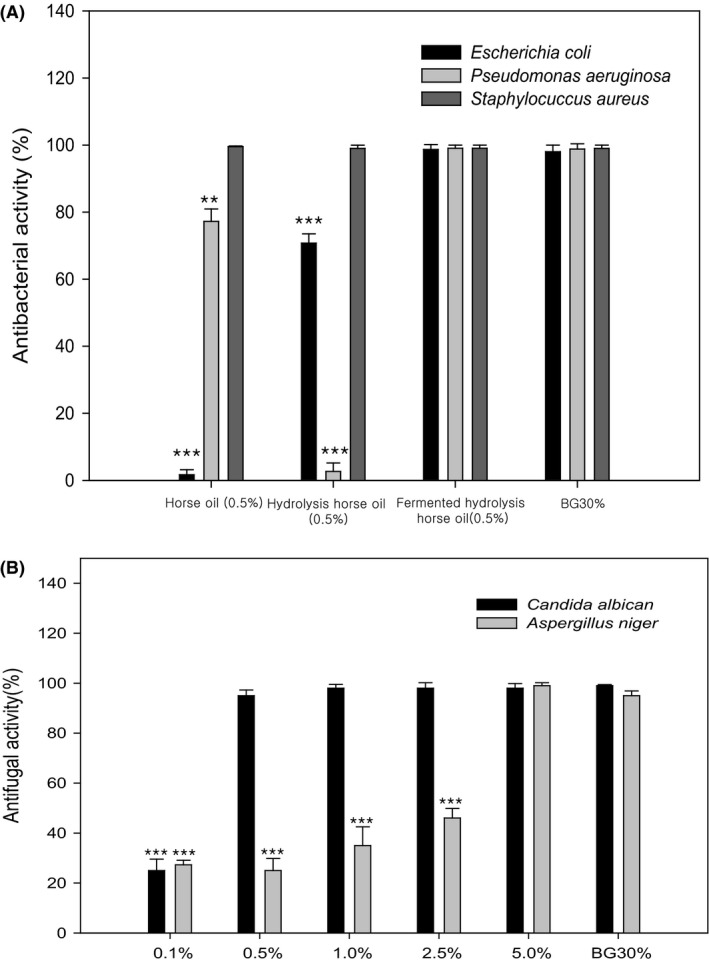
Antibacterial and antifungal activity of horse oil, hydrolysed horse oil and sophorolipids measured by inhibition of bacterial or fungal cell growth. The antibacterial capacity of 0.5% horse oil, hydrolysed horse oil or sophorolipids against *E. coli*,* P. aeruginosa* and *S*. *aureus* was examined and compared with that of the 30% butylene glycol (BG) positive control (A). The antifungal capacity of different concentrations of sophorolipids against *C. albicans* and *A. niger* was also examined and compared with that of the 30% BG positive control (B). Data are presented as the mean ± SD from three separate experiments respectively. **P <* *0.01 vs. BG group; ***P <* *0.001 vs. BG group.

Next, we tested whether sophorolipids possessed antibacterial activity. Gram‐negative *Escherichia coli* and *Pseudomonas aeruginosa*, and Gram‐positive *Staphylococcus aureus*, were treated with 0.5% horse oil, hydrolysed horse oil or sophorolipids. Butylene glycol (BG), used as a solvent and moisturiser in cosmetic products, has antibacterial and antifungal effects (Kinnunen and Koskela, 1991). We, therefore, used 30% BG as a positive control in this study. These reagents were dissolved in deionised water, and as shown in Fig. 6A, horse oil exhibited antibacterial activity against *P. aeruginosa* and *S. aureus*, but not *E. coli*, whereas hydrolysed horse oil inhibited *E. coli* and *S. aureus* but not *P. aeruginosa*. By contrast, sophorolipids displayed very high antibacterial activity against all three bacterial species, in line with the BG positive control (Fig. 6A).

In addition, sophorolipids exhibited antifungal activity against *C. albicans* and *Aspergillus niger* in a concentration‐dependent manner (Fig. 6B). Thus, the five most common microbes found in cosmetic products were efficiently inhibited by sophorolipids, even at a concentration of 0.5%, representing a ~24‐fold increase in activity compared with the 30% BG positive control.

Fermentative production of sophorolipids depends primarily on available carbon sources. Lipids and fatty acids with carbon chains of differing length can be used as carbon sources to produce a variety of sophorolipids, many of which fall into lactonic or acidic sophorolipid classes (Fig. S1). Lactonic sophorolipids are widely applied in fungistats and possess antibacterial activity (Shah *et al*., 2005; Ashby *et al*., 2008). By contrast, acidic sophorolipids have free carboxylic acid groups that make them more soluble. Additionally, lower critical micelle concentrations result in the wide use of acidic sophorolipids in the detergent industry (Hu and Ju, 2001; Delbeke *et al*., 2016).

Compared with chemically synthesised surfactants that are often toxic and difficult to biodegrade, and can cause side effects in humans and the wider environment after long‐term exposure, biosurfactants such as sophorolipids have lower toxicity and higher biodegradability (Lourith and Kanlayavattanakul, 2009). Sophorolipids are particularly biodegradable, and are used widely in cosmetics and personal care products as emulsifying, foaming and wetting agents, as well as solvents, due to their excellent moisture‐retaining capacity (Brown, 1991; Reis *et al*., 2009; Ramrakhiani and Chand, 2011; Goswami *et al*., 2013; Koh *et al*., 2016). Their emulsification and antibacterial properties make sophorolipids suitable for use in antidandruff shampoos, acne removers and deodorants (Otto *et al*., 1999; Hardin *et al*., 2007; Morya *et al*., 2013; Borsanyiova *et al*., 2016), and even in kitchen supplies and detergents (Van Bogaert *et al*., 2013).

## Conclusions

In this study, we established an efficient protocol for generating sophorolipids from hydrolysed and fermented horse oil. By adding an additional 3–4% glucose and maintaining the pH between 4.0 and 4.5 during fed‐batch fermentation, the conversion rate was increased to 71.7 g l^−1^ sophorolipids from hydrolysed horse oil. The final product from fermentation of hydrolysed horse oil was composed of ~40% lactonic diacetyl C18:1, ~23% lactonic diacetyl C18:0 and ~18% acidic diacetyl C18:1 sophorolipids, along with other forms at lower concentrations. Evaluation of biological function revealed a concentration‐dependent stimulation of Col‐I mRNA expression in cultured human fibroblasts, and a relatively high inhibition of elastase (IC_50_ = 38.5 μg ml^−1^). Furthermore, horse oil sophorolipids did not display cytotoxicity at a concentration of 0.5–50 μg ml^−1^, and they stimulated wound healing‐related cell migration, inhibited LPS‐stimulated inflammatory cytokine gene expression in cultured macrophage cells and exhibited strong antibacterial action.

## Experimental procedures

### General

*Candida bombicola* cells were stored in 50% glycerol at −70°C, and cultured in YM broth (3.0 g of yeast extract, 3.0 g of malt extract, 5.0 g of peptone and 10.0 g of glucose per l) at 30°C with shaking at 160 rpm for 48 h in a shaking incubator. The culture medium was used to inoculate a 5 l fermenter for large‐scale culturing. Cells were harvested, frozen and stored until needed. Glucose used during culturing was purchased from Sigma‐Aldrich (St. Louis, Missouri, USA), horse oil was purchased from BiolandBiotec. Co., Ltd. and all other ingredients were purchased from Difco or Merck (Detroit, Michigan, USA).

Standard medium for culturing fermented sophorolipids contained 100 g of glucose, 5 g of yeast extract, 1 g of KH_2_PO_4_, 0.5 g of MgSO_4_∙7H_2_O, 0.1 g of CaCl_2_∙2H_2_O, 0.1 g of NaCl and 0.7 g of peptone per l, along with different concentrations of horse oil or hydrolysed horse oil. Hydrolysed horse oil was made from horse oil by lipase treatment. A 5% culture volume was added to the 5 l jar fermenter (Changzhou Sungod Biotechnology and Engineering Equipment Co., Ltd., Changzhou, Jiangsu, China) as an inoculum, and the 1 l culture volume was incubated at 25°C, with an initial pH of 5.5, agitation at 500 rpm, an aeration rate of 1.0 vvm and a cultivation time of 7 days. In the pH 4.0 conditions, an automatic pH control system was used in 3 M NaCl. The 5 l fermenter was fed with 5% horse oil up to day 2 or 3 (10% horse oil). In the case of hydrolysed horse oil, the 5 l fermenter was fed with 5% hydrolysed horse oil up to day 2, 3 or 4 (15% horse oil).

Hydrolysed horse oil was incubated in the 5 l fed‐batch fermenter for 2 days, and the glucose concentration was maintained at ~30–40 g l^−1^. After incubation for 3 days, the pH of the fermentation system was maintained between 4.0 and 4.5. During the fermentation procedure, samples were collected every day to measure the pH, DCW (g l^−1^), crude sophorolipid content (g l^−1^) and glucose level (g l^−1^).

### Purification and structural analysis by UPLC‐MS and NMR

A mixture of sophorolipids was obtained by solvent extraction and dissolved in methylene chloride as solvent. This sample was separated by silica gel chromatography (Merck Co.; mesh = ~230–400, column = 30 × 60 mm) using a methylene chloride/methanol gradient. The main fraction was then re‐chromatographed on silica gel, the eluent solution was evaporated under reduced pressure and the molecular weight and structure were investigated using an ACQUITY UPLC‐Micromass ZQ2000 (Waters, Milford, Massachusetts, USA) and an AV‐600 NMR instrument (Bruker, Germany). The UPLC‐MS eluent solution and mass analysis conditions are summarised in Tables S3 and S4.

### Biological evaluation of newly synthesised sophorolipids

#### Antiwrinkle *in vitro* assays

Using *in vitro* cultured fibroblasts, the potential of the synthesised horse oil sophorolipids to stimulate collagen synthesis and inhibit elastase was tested. Human fibroblasts were cultured at a density of 1 × 10^6^ cells/well in 60 mm plates at 37°C for 24 h. Cultures were then substituted with serum‐free medium and cultured at 37°C for 24 h with and without the tested compounds.

Collagen mRNA expression was examined by real‐time PCR (RT‐PCR). The RNA pellet was isolated from treated and untreated cultured fibroblasts with TRIzol reagent (Invitrogen, Carlsbad, California, USA). A mixture was prepared using a high‐capacity RNA‐to‐cDNA kit (Applied Biosystems), and 1 μg of RNA was added to each sample and incubated at 37°C for 60 min followed by 95°C for 5 min to synthesise cDNA. A primer mixture was prepared using SYBR green PCR mix (Applied Biosystems). RT‐PCR (Applied Biosystems) was performed with an initial holding period at 50°C for 20 s, denaturation at 95°C for 15 s and annealing at 60°C for 1 min, followed by 40 cycles at 95°C for 30 s and 60°C for 15 s. Sequences of primers used to amplify collagen I (Col‐I) and the β‐actin reference gene are listed in Table 2.

**Table 2:** Real‐time PCR primer sequences

Substance	Base sequence (5′→3′)
Collagen type 1 (Col‐1)	
Forward	5′‐AGCAAGAACCCCAAGGACAA‐3′
Reverse	5′‐CGAACTGGAATCCATCGGTC‐3′
β‐Actin	
Forward	5′‐GGCACCCAGCACAATGAAG‐3′
Reverse	5′‐CCGATCCACACGGAGTACTTG‐3′
tnf‐α	
Forward	5′‐TCT CAT CAG TTC TAT GGC CCA GA‐3′
Reverse	5′‐CAG GCT TGT CAC TCG AAT TTT G‐3′
COX‐2	
Forward	5′‐GGC CAT GGA GTG GAC TTA AAT C‐3′
Reverse	5′‐AAG GCG CAG TTT ATG TTG TCT GT‐3′
il‐6	
Forward	5′‐TCG GCA AAC CTA GTG CGT TAT‐3′
Reverse	5′‐TTT CTG ACC ACA GTG AGG AAT GTC‐3′
GAPDH	
Forward	5′‐GGC ATC TTG GGC TAC ACT GAG‐3′
Reverse	5′‐GGA AGA GTG GGA GTT GCT GTT G‐3′

To measure elastase inhibition, hydrolysed horse oil and synthesised sophorolipids were prepared in 0.267 M TRIS buffer adjusted to pH 8 with 0.267 M HCl. Substrate (Succ‐Ala‐Ala‐Ala‐p‐nitroanilide; Sigma) was prepared at a final concentration of 8.8 mM, and enzyme (porcine pancreatic elastase) was prepared at 10 μg ml^−1^. Control samples containing 100 μl of distilled water were used as a blank (absorbance expressed as A). A no‐enzyme control containing 120 μl of DW was also included (absorbance expressed as D). A 60 μl sample of 0.1 M potassium phosphate was used as buffer solution (pH 6.8), to which was added 20 μl of substrate at varying concentrations. Samples were mixed with 20 μl of enzyme and incubated at 25°C for 15 min. The produced *p*‐nitroaniline was quantified at a wavelength of 410 nm (absorbance expressed as B). A 60 μl sample of buffer solution, 20 μl of substrate, 20 μl of DW and 100 μL of sample were mixed and treated as described above (absorbance expressed as C). IC_50_ values (expressed in μg ml^−1^) were determined to estimate the elastase inhibition activity using the following formula: Inhibition of Elastase (%) = [1−[(B−C)/(A−D)]] × 100.

#### Cell cytotoxicity test (MTT assay)

Fibroblasts (ATCC) were plated at a density of 1 × 10^5^ cells/well in 24 well plates and cultured for 24 h at 37°C. Medium was changed to serum‐free medium, and cells were cultured with different concentrations of samples at 37°C for a further 24 h. MTT (M5655; Sigma‐Aldrich) was diluted 10‐fold and added to each well, and culturing continued for 4 h at 37°C. The medium was then aspirated, 1 ml of dimethyl sulfoxide (DMSO) was added to each well and the supernatant was quantified using a Tecan Infinite M200 enzyme‐linked immunosorbent assay reader (Tecan, Mannedorf, Meilen District, Swiss Austria) at a wavelength of 570 nm. Results are expressed as the percentage optical density relative to the negative control.

#### *In vitro* wound‐healing assay (cell migration test)

The composition of the skin surface changes as epidermis cells slough continuously while healthy cells take their place from beneath the epidermis. When cells on the outer surface are restored, the basement membrane is exposed, and wounds on the skin surface become red and eventually disappear. Consequently, new reticular tissue forms new blood vessels and new collagen. Fibroblasts are crucial for the formation of collagen and other connective tissues, and are therefore essential for wound repair. When inflammation occurs, blood platelets and macrophagocytes are increased, and reaction with growth factors initiates wound healing. Initially, small quantities of collagen alone fill the wound, and collagen synthesis and epidermis cell movement occur concurrently. This process was investigated using an Oris Cell migration assay kit, and cell migration was assessed by microscopy. Fibroblasts were cultured at a density of 1 × 10^4^ cells/well in 96 well plates at 37°C for 24 h, medium was replaced with serum‐free medium and culturing continued at 37°C for 6 days. Cells were washed with 1 × phosphate‐buffered saline (PBS) prior to observation by microscopy.

#### Anti‐inflammatory activity

Raw 264.7 cells were plated at a density of 3 × 10^6^ cells/well in a 60 mm plate and incubated for 24 h at 37°C. The medium was then changed to medium containing 0.1 μg ml^−1^ LPS (Sigma) and culturing continued with and without different concentrations of horse oil, hydrolysed horse oil or sophorolipids. The medium was then aspirated, cells were washed with PBS, 1 mL of TRIzol (Invitrogen) was added and cells were centrifuged at 15 000 rpm for 30 min at 4°C. The supernatant was mixed with 2‐propanol and centrifuged at 15 000 rpm for 20 min at 4°C, and this step was repeated. The substrate was mixed with 1 ml of 75% EtOH and centrifuged at 13 000 rpm at 4°C. The resulting RNA pellet was air‐dried at room temperature to remove ethanol, dissolved in diethyl pyrocarbonate (DEPC)‐treated DW and quantified using a Qubit system (Invitrogen). A mixture was prepared using an RNA‐to‐cDNA kit (Applied Biosystems), 1 μg of RNA was added to each sample and a PCR system (Invitrogen) was used to synthesise cDNA at 37°C for 60 min followed by 95°C for 5 min. A mixture was prepared using SYBR green PCR mix (Applied Biosystems). RT‐PCR (Applied Biosystems) was performed as follows: an initial holding period at 50°C for 20 s, denaturation at 95°C for 15 s and annealing at 60°C for 1 min, followed by 40 cycles at 95°C for 30 s and 60°C for 15 s. Sequences of primers used to amplify TNF‐α, COX‐2, IL‐6 and glyceraldehyde 3‐phosphate dehydrogenase (GAPDH) are listed in Table 2.

#### Antibacterial and fungal tests

Antibacterial activity of horse oil, hydrolysed horse oil and synthesised sophorolipids was determined against *E. coli*,* P. aeruginosa* and *S. aureus*. Bacterial cells were cultured in Tryptic Soy Broth (Difco) at 30°C with shaking at 160 rpm for 24 h in flasks. *C. albicans* cells were cultured in YM broth (Difco) at 25°C with shaking at 160 rpm for 48 h, and *A. niger* cells were cultured in Potato Dextrose Agar at 25°C for 7 days. After static culturing, black spores were sampled and antibacterial tests were conducted. The above five strains were inoculated with 0.5% horse oil, 0.5% hydrolysed horse oil, 0.5% fermented hydrolysed horse oil (sophorolipids) or 30% 1,3‐buthylene glycol at a cell density of 1.0 × 10^5 ^CFU ml^−1^ and incubated for 24 h to calculate cell survival. Antibacterial activity during the early stages of cultivation was compared after 1 day, and the reduction in the survival of bacteria was expressed as a percentage.

### Statistical analysis

For experiments leading to results shown in Figs 1, 3, 5 and 6, at least three separate experiments were performed and data are expressed as means ± standard deviation (SD). Comparison of different groups was performed by one‐way ANOVA, followed by *post‐hoc* pairwise repetitive comparisons with Tukey tests. Statistical analysis was performed with Origin 8.6 software, and *P *<* *0.05 was considered statistically significant.

## Conflict of Interest

None declared.

## Ethical approval

This article does not contain any studies with human participants or animals performed by any of the authors. Horse oil was purchased from Shijiazhuang China Cosmetics Trade Co. Ltd.

## Supporting information


**Fig. S1.** The sophorolipid structure of acidic form and lactonic form (R=H or Acetyl group).
**Fig. S2.** UPLC‐Mass total flow chart.
**Fig. S3.** (A) 1H‐NMR spectrum of lactonic sophorolipid(diacetyl, 18:1) (MeOD, 600MHz).
**Fig. S4**. COSY spectrum of lactonic sophorolipid (diacetyl, 18:1).
**Fig. S5.** DEPT spectrum of lactonic sophorolipid (diacetyl, 18:1).
**Fig. S6**. HMBC spectrum of lactonic sophorolipid (diacetyl, 18:1).
**Table S1.** Comparisons of saturated and unsaturated fatty acids composition ratio in human skin with horse oil.
**Table S2.** Analysis of molecular weights of sophorolipids.
**Table S3.** Eluent solvent conditions of UPLC‐Mass (A Solvent is 90% acetonitrile; B Solvent is 10 mM ammonium acetate solution).
**Table S4**. Analysis condition of UPLC‐Mass.
