# Supplementary material for: A novel microbial technique for producing high‐quality sophorolipids from horse oil suitable for cosmetic applications
Source: Microb Biotechnol. 2018 Jul 18;11(5):917–29. doi: 10.1111/1751-7915.13297 (PMC6116743; doi:10.1111/1751-7915.13297)
Supplement: Supplementary file 1 — Fig. S1. The sophorolipid structure of acidic form and lactonic form (R=H or Acetyl group). Fig. S2. UPLC‐Mass total flow chart. Fig. S3. (A) 1H‐NMR spectrum of lactonic sophorolipid(diacetyl, 18:1) (MeOD, 600MHz). Fig. S4. COSY spectrum of lactonic sophorolipid (diacetyl, 18:1). Fig. S5. DEPT spectrum of lactonic sophorolipid (diacetyl, 18:1). Fig. S6. HMBC spectrum of lactonic sophorolipid (diacetyl, 18:1). Table S1. Comparisons of saturated and unsaturated fatty acids composition ratio in human skin with horse oil. Table S2. Analysis of molecular weights of sophorolipids. Table S3. Eluent solvent conditions of UPLC‐Mass (A Solvent is 90% acetonitrile; B Solvent is 10 mM ammonium acetate solution). Table S4. Analysis condition of UPLC‐Mass. [file MBT2-11-917-s001.doc]

**A novel microbial technique for producing high-quality sophorolipids from horse oil suitable for cosmetic applications**

Yoojae Maeng ^1,2＃^, Kyoung Tae Kim ^1,2＃^, Xuan Zhou ^3＃^, Litai Jin ^1,2^, Ki Soo Kim ^1,4^, Young Heui Kim ^5^, Suyeon Lee ^5^, Ji Ho Park ^6^, Xiuyu Chen ^4^, Mingxia Kong ^4^, Lu Cai ^1,7^,*, Xiaokun Li ^1,2^,*

1. *School of Pharmaceutical Sciences, Wenzhou Medical University, Wenzhou 325035, China*
2. *Collaborative Innovation Center of Biomedicine, Wenzhou Medical University-Wenzhou University, Wenzhou 325035, China*
3. *Ningbo First Hospital, Ningbo, 315000, China*
4. *BiolandBiotec. Co., Ltd., Zhangjiang Modern Medical Device Park, Pudong, Shanghai 201203, China*
5. *SK Bioland, 59, Songjeongni 2-gil, Byeongchen, Dongnam, Cheonan, Chungnam 31257, Korea*
6. *SK Bioland, 162, Gwahaksaneop 3-ro, Ochang, Cheongwon, Cheongju, Chungbuk 28125, Korea*
7. *Pediatric Research Institute, Departments of Pediatrics, Radiation Oncology, Pharmacology and Toxicology, University of Louisville, Louisville, KY 40202, USA*

^＃^These authors contributed equally to this work

* Corresponding author

Dr. Xiaokun Li at School of Pharmaceutical Sciences, Wenzhou Medical University, Chashan University-Town, Wenzhou, Zhejiang 325035, China. Tel: 86-577-86699350, Fax: 86-577-86699238, Email: [lixk1964@163.com](mailto:lixk1964@163.com);

or Dr. Lu Cai, Tel: 001 502-8522214, Fax: 502-8525634, Email: [lu.cai@louisville.edu](mailto:lu.cai@louisville.edu)

**Supplemental Tables**

**Supplemental Figures**

**Supplemental Tables**

**Table S2. Analysis of molecular weights of sophorolipids**

**___________________________________________________________________________**

**Sophorolipids R.T.(min) M.W. [M+NH4]^+^ Contents (%)**

**___________________________________________________________________________**

Lactonic form (Diacetyl, C18:0) 7.26; 7.57 690 708 22.78

Acidic form (Diacetyl, C18:1) 4.52; 4.55; 4.61 706 724 17.69

Lactonic form (Monoacetyl, C18:1) 5.87; 6.09 646 664 8.14

Lactonic form (Diacetyl, C18:1) 7.07 688 706 40.12

Acidic form (Diacetyl, C18:2) 4.33 704 722 4.15

Lactonic form (Diacetyl, C18:2) 6.17; 6.19 686 704 6.76

Acidic form (Diacetyl, C18:3) 7.19; 7.22 702 720 2.83

__________________________________________________________________________________

**Table S3.** Eluent solvent conditions of UPLC-Mass (A Solvent is 90% acetonitrile; B Solvent is 10 mM ammonium acetate solution).

**
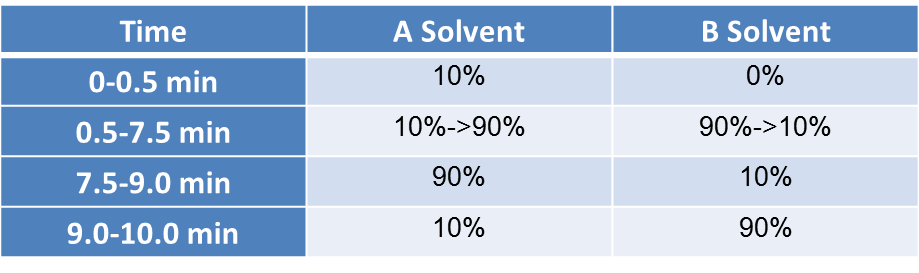
**

**Table S4**. Analysis condition of UPLC-Mass

**
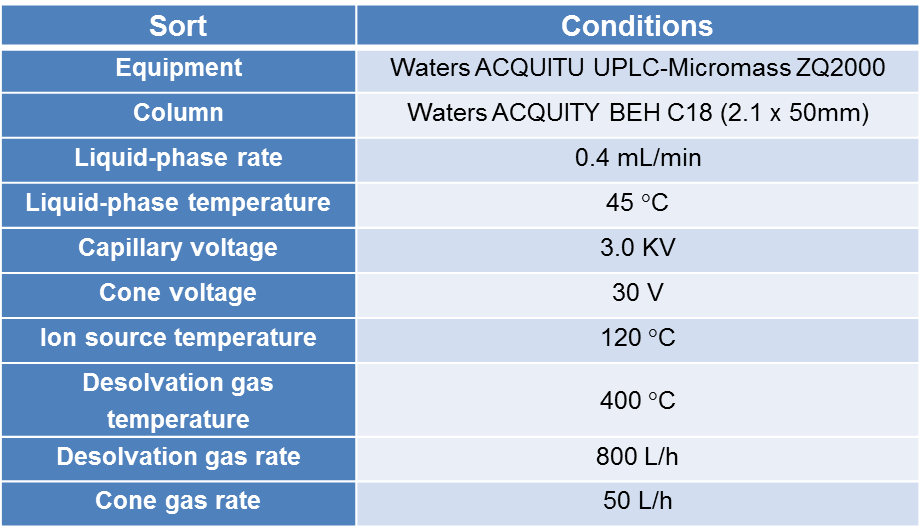
**

**Supplemental Figures**

A

B


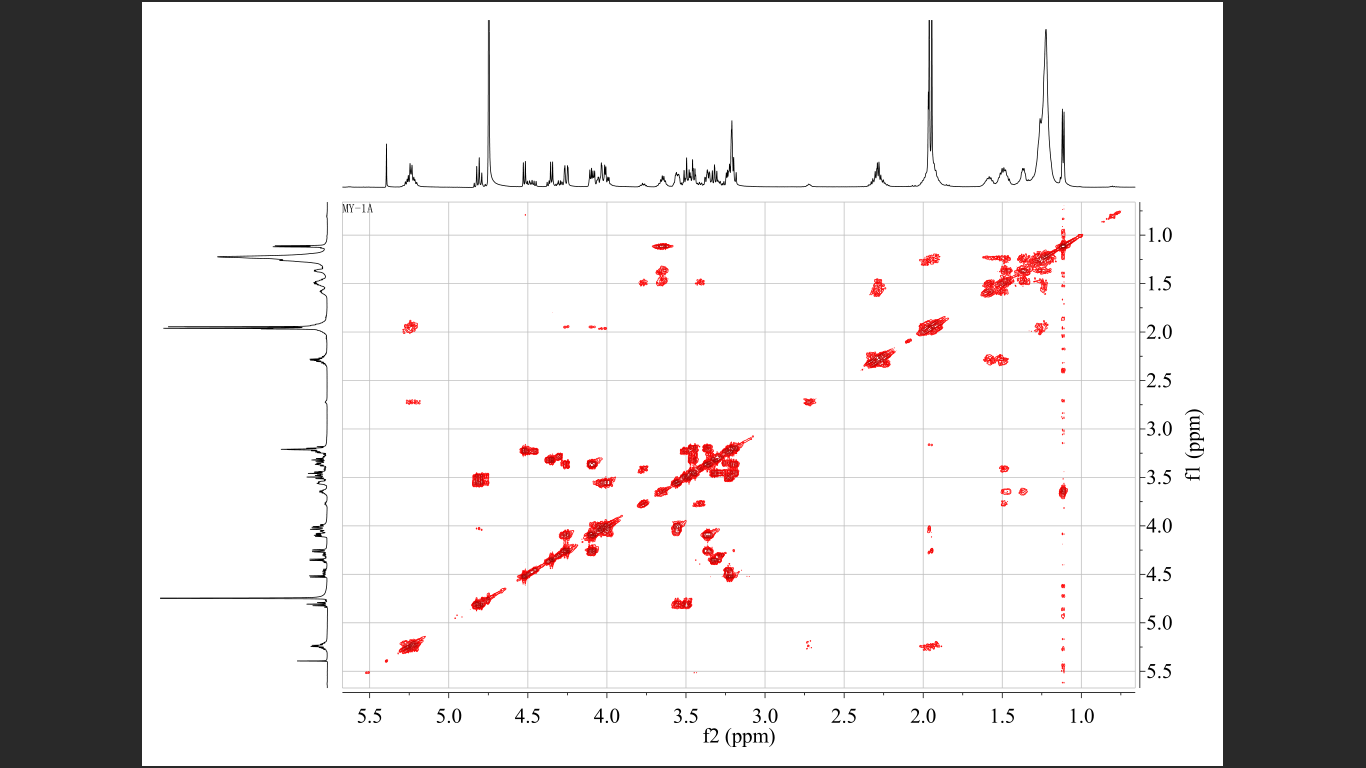


**Figure S4**. COSY spectrum of lactonic sophorolipid (diacetyl, 18:1)


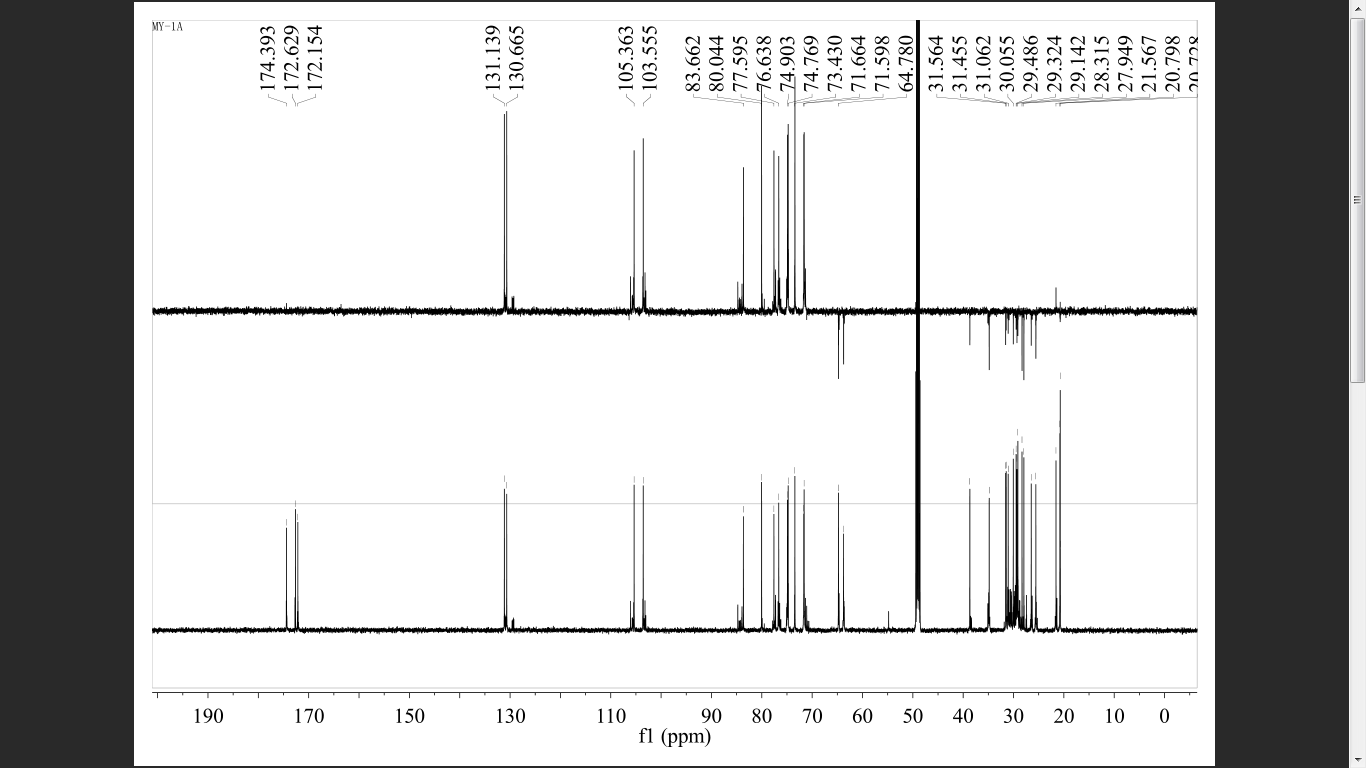


**Figure S5.** DEPT spectrum of lactonic sophorolipid (diacetyl, 18:1)


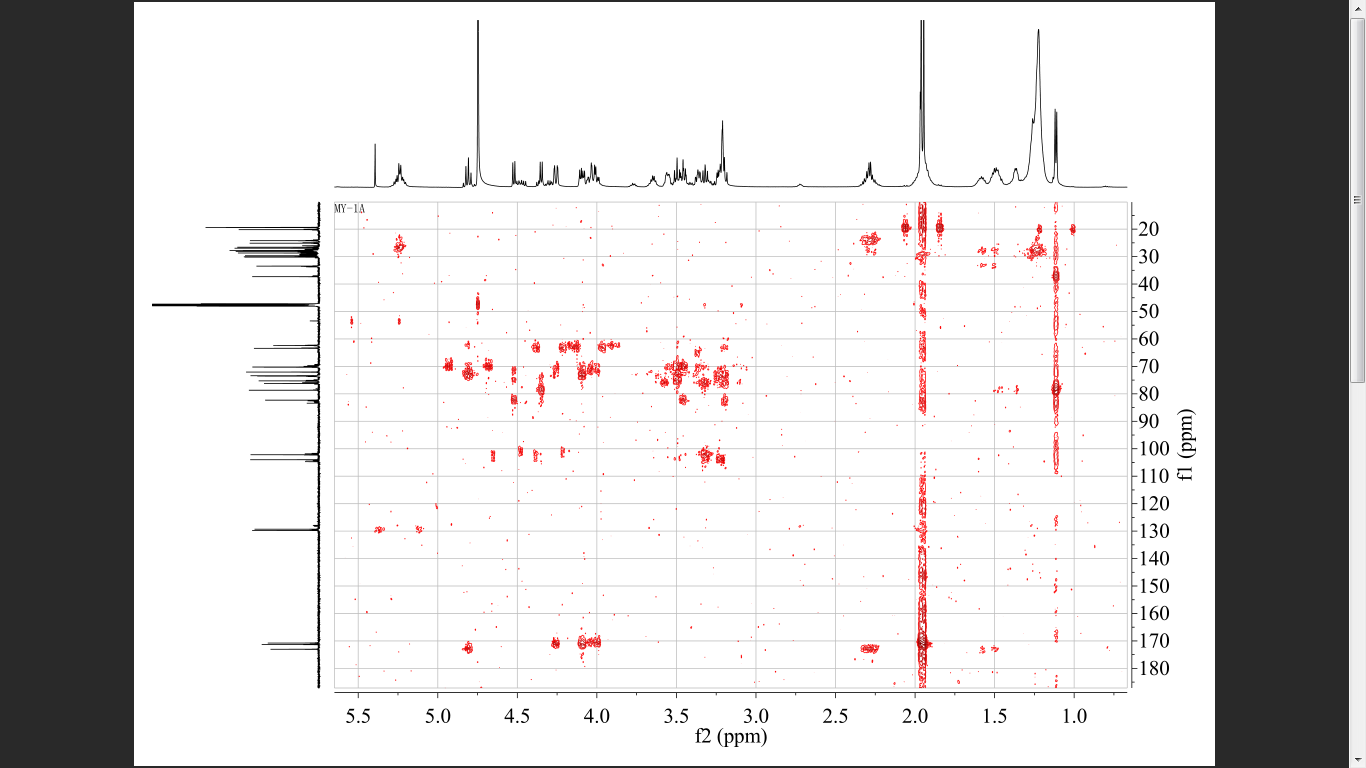


**Figure S6**. HMBC spectrum of lactonic sophorolipid (diacetyl, 18:1)
